# Treadmill Exercise Ameliorates Adult Hippocampal Neurogenesis Possibly by Adjusting the APP Proteolytic Pathway in APP/PS1 Transgenic Mice

**DOI:** 10.3390/ijms22179570

**Published:** 2021-09-03

**Authors:** Haizhen Yu, Chenfei Zhang, Jie Xia, Bo Xu

**Affiliations:** 1 Key Laboratory of Adolescent Health Assessment and Exercise Intervention of Ministry of Education, East China Normal University, Shanghai 200241, China; pzghgzy@126.com (H.Y.); 52201000015@stu.ecnu.edu.cn (C.Z.); jxia@tyxx.ecnu.edu.cn (J.X.); 2 School of Physical Education and Health Care, East China Normal University, Shanghai 200241, China

**Keywords:** Alzheimer’s disease, treadmill exercise, hippocampal microenvironment, hippocampal neurogenesis, APP proteolytic pathway

## Abstract

Alzheimer’s disease (AD) is a neurodegenerative disorder known to cause cognitive impairment among the elderly worldwide. Although physical exercise-induced adult hippocampal neurogenesis (AHN) improves cognition, understanding its underlying molecular mechanisms requires further investigation using AD mouse models. In this present work, we subjected amyloid precursor protein (APP)/PS1 mice to a 12-week aerobic treadmill exercise to investigate AHN and its potential mechanisms. We divided 3-month-old littermates wild-type and APP/PS1 transgenic male mice into four groups, and the exercise groups performed 12-week treadmill exercise. Next, we evaluated the influence of treadmill exercise on learning and memory capacity, AHN, and APP proteolytic pathway-related factors. As per our results, the treadmill exercise was able to improve the hippocampal microenvironment in APP/PS1 mice probably by regulating various neurotrophic factors and secretases resulting in APP cleavage through a non-amyloidogenic pathway, which seems to further promote new cell proliferation, survival, and differentiation, enhancing hippocampal neurogenesis. All of these effects ameliorate learning and memory capacity. This study provides a theoretical and experimental basis for understanding AHN in an AD mouse model, which is beneficial for preventing and treating AD.

## 1. Introduction

Alzheimer’s disease (AD) is known to be a prevalent chronic neurodegenerative disease affecting people older than 65; it is often characterized by progressive memory loss and cognitive decline [1]. β-Amyloid (Aβ) deposition, neurofibrillary tangle (NFT) formation, and neuroinflammation are the main hallmarks of AD [2,3]. Despite substantial advances in understanding AD’s cellular and molecular mechanisms, the current therapies aimed at reducing Aβ levels have failed to effectively prevent disease onset and progression [4]. One of the clinical manifestations of AD is the death of a large number of neurons, which causes neurocircuit alteration and neurocognitive dysfunction. In this case, treatments aiming for neuronal restoration and regeneration might emerge as a potential therapeutic approach to counteract AD progression [4,5].

The steps of adult hippocampal neurogenesis (AHN) are the division of neural stem cells (NSCs), the maturation of neural progenitor cells (NPCs), and their migration and maturation into neurons, which are ultimately incorporated into hippocampal neuronal networks [6,7]. AHN is the regenerative capacity of the adult brain and a remarkable form of hippocampal plasticity [8]. A number of behavioral experiments on spatial recognition and memory have already demonstrated that AHN plays a crucial role in learning and memory maintenance [9,10,11]. Numerous extrinsic and intrinsic factors regulate the dynamic AHN process in the mammalian brain [6,8,12]. Many reports indicate that physical exercise promotes neural functions by regulating the proliferation, differentiation, survival, and maturation of NPCs, thus confirming the positive relationship between exercise-induced cognition improvement and hippocampal neurogenesis [8,13,14,15,16]. Thus, the fact that physical exercise improves cognition by enhancing hippocampal neurogenesis has captured the attention of many neuroscientists. Furthermore, a conserved type I membrane protein, the amyloid precursor protein (APP), was also determined to significantly influence AHN. APP metabolites such as soluble APP (sAPP), sAPP-α, sAPP-β, Aβ peptides, and the APP intracellular C-terminal domain (AICD) seem to regulate different functions in NSCs, including proliferation, gliogenesis, neurogenesis, or apoptosis [6,17].

However, some research works related to AHN in AD animal models remain controversial. When studying hippocampal neurogenesis in APP transgenic mice, Haughey et al. [18] found that new cells in the hippocampal dentate gyrus (DG) region of APP transgenic mice had reduced proliferation and differentiation capacity. Meanwhile, Mirochnic et al. [19] found that APP23 transgenic mice displayed significantly faster cell proliferation than wild-type mice in the subgranular zone. Preventing hippocampal neurogenesis in healthy rats by focal irradiation causes cognitive deficits, and 3 weeks’ worth of wheel running reduces the impairment [20]. Nevertheless, another study proved that using focal irradiation to inhibit AHN only destroys contextual fear conditioning and synaptic plasticity in the DG and has no influence on spatial learning and cognitive ability in the Morris water maze test [21]. Several studies have demonstrated that decreasing Aβ plaque levels increases AHN [22], while Michael et al. discovered that exposing cells to extrinsic Aβ and other amyloids is insufficient to impair AHN [23].

Moreover, the precise molecular mechanisms of AHN induction by physical exercise remain to be largely unclear, and the effect of APP on neurogenesis under physical exercise conditions has not been fully explored. The microenvironments of the subgranular zone are essential in supplying and modulating fate-determining cues in the adult hippocampus [24]. Progenitors, mature neurons, oligodendrocytes, and astrocytes cells might all provide some elements regulating exercise-induced AHN in the microenvironments [8,25]. Furthermore, the expression of various neurotrophic factors and key secretases in the hippocampus microenvironments might alter the APP proteolytic pathway. APP is related to memory processes, neurite outgrowth, synaptic formation, and neuronal survival, protection, and plasticity [26,27,28]. APP knockout mice thus display behavioral and cognitive deficits [29]. Confusingly, mice overexpressing APP also present learning and memory impairments [30]. Thus, understanding the effects of physical exercise on AHN and the relevant mechanisms linked to the APP proteolytic pathway requires further exploration.

In this study, we document the effects of aerobic exercise on learning and memory capacity, AHN, and APP proteolytic pathway-related factors in APP/PS1 mice to evaluate whether the exercise-induced APP proteolytic pathway promotes AHN by affecting the hippocampal microenvironment. Our results have uncovered the molecular mechanism of exercise-induced AHN, which might provide a theoretical and experimental basis for the pathogenesis, prevention, and treatment of AD.

## 2. Results

### 2.1. Treadmill Exercise Enhanced Learning and Memory Capacity in APP/PS1 Transgenic Mice

**Figure 1:**
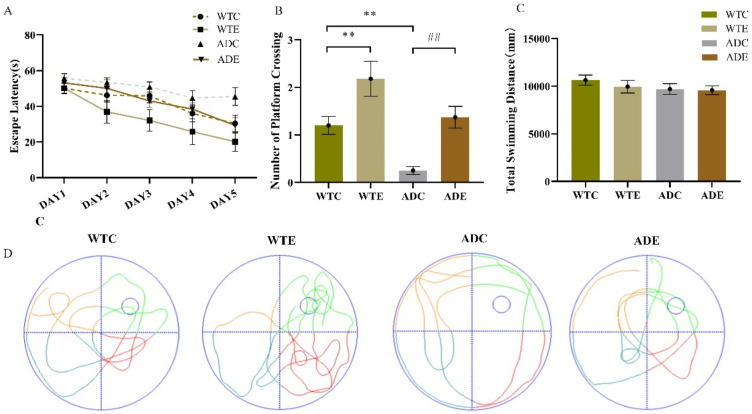
Effect of treadmill exercise on spatial learning and memory in mice. (**A**) Escape latency in the place navigation test. (**B**) Number of platform crossings in the spatial probe test. (**C**) Total swimming distance in the spatial probe test. (**D**) Swimming paths in the spatial probe test. *n* = 6 per group. ** *p* < 0.01 compared with WTC. ## *p* < 0.01 compared with ADC.

The results in this study were consistent with our previous publications [31,32]. In the navigation test, as shown in Figure 1A, the escape latency was noted to decrease from the 2nd to the 5th day in all groups, but it decreased less sharply in the ADC group than in the others. Treadmill exercise significantly reduced the escape latency, especially on the 5th day, demonstrating that the ADE group had a significantly better learning ability than the ADC group. We assessed memory capacity by counting the platform position crossings. Figure 1B shows that exercise significantly increased the number of platform position crossings (WTC vs. WTE, *p* < 0.01; ADC vs. ADE, *p* < 0.01), and the ADC group crossed the platform position much fewer times than the WTC group (*p* < 0.01). As presented in Figure 1C, all the groups had similar total swimming distances, illustrating that cognitive dysfunction, rather than swimming capacity, accounted for the differences. Figure 1D shows the swimming trajectories of mice in the absence of the platform for 60 s (spatial probe trial). These behavior tests suggest that APP/PS1 transgenic mice had notably impaired spatial learning and memory, and aerobic treadmill exercise alleviated these deficiencies.

### 2.2. Treadmill Exercise Decreased Aβ Plaque Levels and Apoptosis in APP/PS1 Transgenic Mice

**Figure 2:**
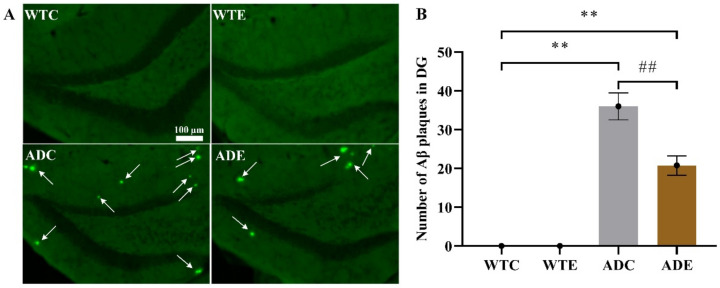
Effect of treadmill exercise on Aβ deposition in mice. (**A**) Aβ thioflavin-S staining in the hippocampus. (**B**) Aβ plaque quantification in the hippocampus. *n* = 4 for each group. ** *p* < 0.01 compared with WTC. ## *p* < 0.01 compared with ADC. The arrows point to the Aβ plaques.

**Figure A1:**
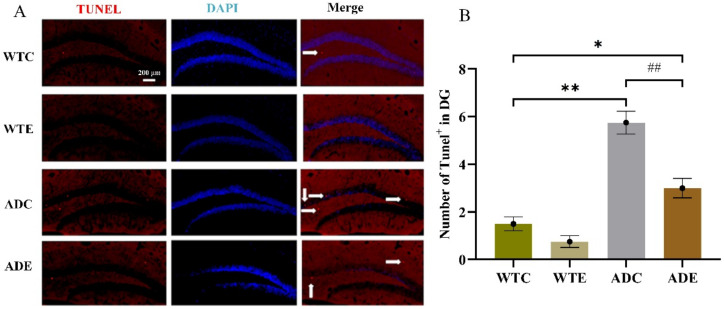
Effect of treadmill exercise on cell apoptosis in mice. (**A**) TUNEL staining results in hippocampus. (**B**) The number of cell apoptosis in hippocampus. *n* = 4 for each group. * *p* < 0.05, ** *p* < 0.01 compared with WTC. ## *p* < 0.01 compared with ADC. The arrows point to the apoptosis cells.

To determine the pathological features underlying the behavior characteristics demonstrated above in APP/PS1 transgenic mice, we quantified Aβ plaque in the hippocampus by thioflavin-S staining (Figure 2). Two-way ANOVA indicated that both genotype (F(1,12) = 175.1, *p* < 0.0001) and exercise (F(1,12) = 12.64, *p* = 0.0040) as well as their interaction [F(1,12) = 12.64, *p* = 0. 0040] significantly affected Aβ levels. As per the post-hoc analysis, it was determined that the ADC group had remarkably higher Aβ plaque than the WTC group (*p* < 0.01), but 3 months of treadmill exercise significantly alleviated this increase in ADE mice compared with ADC ones (*p* < 0.01). These results indicate that the APP/PS1 transgenic mice indeed had higher Aβ deposition, which was significantly reduced by treadmill exercise. Moreover, many more cells died in AD mice, especially in ADC mice, than in WTC (*p* < 0.01) and ADE mice (*p* < 0.01), as shown in the Appendix A Figure A1. Both genotype (F(1,12) = 78, *p* < 0.0001) and exercise (F(1,12) = 22.62, *p* = 0.0005), as well as their interaction (F(1,12) = 7.385, *p* = 0.0187), have significantly affected apoptosis. These AD hallmarks were consistent with the behavior observed in the AD mice.

### 2.3. Effects of Treadmill Exercise on Hippocampal Neurogenesis

This study aimed to determine the effects of aerobic exercise on AHN in APP/PS1 transgenic mice and to further investigate the molecular mechanism in relation to the APP proteolytic pathway. AHN is a dynamic process including the proliferation, differentiation, survival, and maturation of NPCs, which we thoroughly investigated as follows.

#### 2.3.1. Treadmill Exercise Improved the Proliferation of NSCs

**Figure 3:**
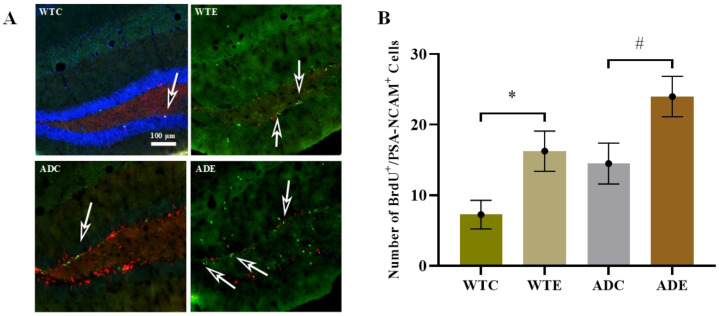
Effect of treadmill exercise on NSC proliferation in mice. (**A**) Fluorescence staining images of BrdU/PSA-NCAM-positive cells. The green, red and blue fluorescence represents the positive features of BrdU, PSA-NCAM and DAPI respectively. (**B**) Number of BrdU/PSA-NCAM-positive cells. *n* = 4 for each group. * *p* < 0.05 compared with WTC. # *p* < 0.05 compared with ADC. The arrows point to the BrdU/PSA-NCAM co-localization-positive cells.

**Figure A2:**
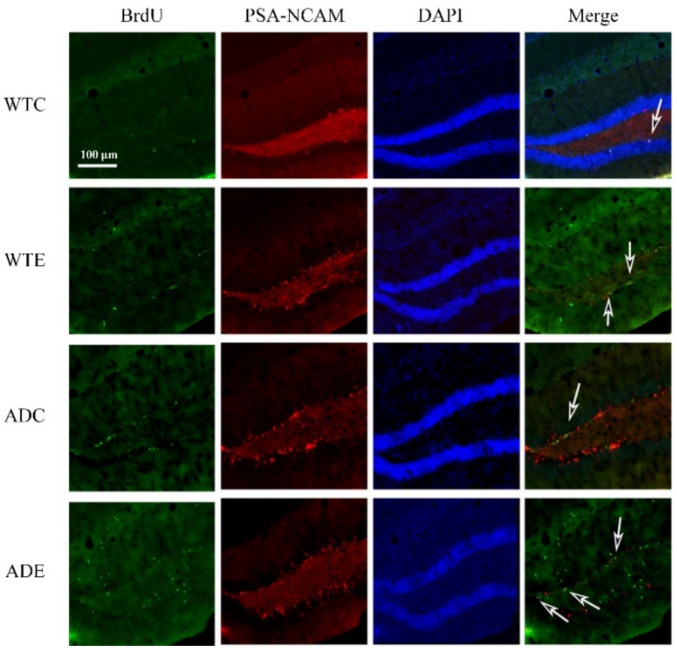
Effect of treadmill exercise on NSC proliferation in mice. Fluorescence staining images of BrdU/PSA-NCAM-positive cells. The arrows point to the BrdU/PSA-NCAM co-localization-positive cells.

After 12 weeks of exercise, we prepared four mice in each group to determine the proliferation of new cells using the double immunofluorescence BrdU/PSA-NCAM co-localization method (Figure 3). Appendix A Figure A2 presents further information about fluorescence staining images. Two-way ANOVA indicated that both genotype (F(1,12) = 7.837, *p* = 0.0161) and exercise (F(1,12) = 11.92, *p* = 0.0048), but not their interaction (F(1,12) = 0.0087, *p* = 0.9272), significantly affected proliferation. The post-hoc analysis revealed that APP/PS1 transgenic mice had more BrdU/PSA-NCAM co-localization-positive cells than wild-type mice. Treadmill exercise significantly increased the number of proliferating cells in both APP/PS1 transgenic and wild-type mice (*p* < 0.05). These results demonstrated that APP and PS1 overexpression in AD mice promoted NSC proliferation, and treadmill exercise promoted it further.

#### 2.3.2. Treadmill Exercise Promoted Cell Survival

**Figure 4:**
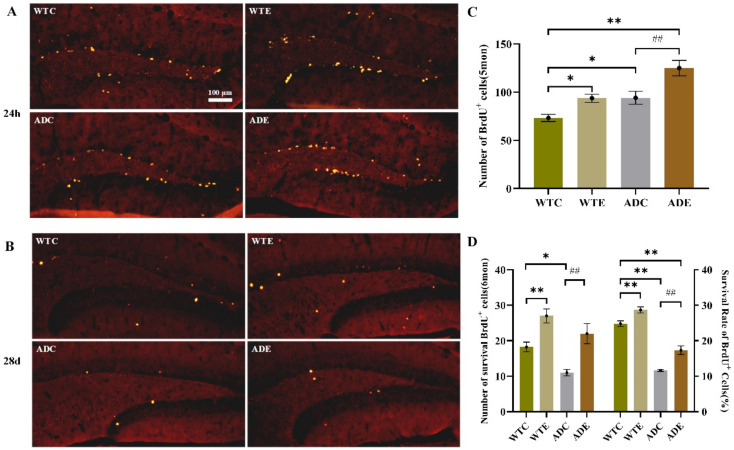
Effect of treadmill exercise on the survival of new cells in mice. (**A**) Fluorescence staining images of BrdU-positive cells in 5-month-old mice 24 h after BrdU injection. (**B**) Fluorescence staining images of BrdU-positive cells in 6-month-old mice 28 days after BrdU injection. (**C**) Number of cells that had survived in 5-month-old mice 24 h after BrdU injection. (**D**) Number of cells that had survived in 6-month-old mice 28 days after BrdU injection (left); Survival rate of cells in 5-month-old mice 28 days after BrdU injection (right). *n* = 4 for each group. * *p* < 0.05, ** *p* < 0.01 compared with WTC. ## *p* < 0.01 compared with ADC.

We evaluated how exercise affected the survival of the progeny of NPCs by staining BrdU-positive cells 28 days and 24 h after the last BrdU injection at the 8th week (Figure 4A,B). We defined survival as the ratio of BrdU-positive cells at 28 days to that at 24 h. Figure 4 shows that the number of BrdU-positive cells was significantly higher at 24 h than at 28 days, indicating that many new cells died. This result was consistent with those of the apoptosis assay shown in the Appendix A Figure A1. Although APP/PS1 transgenic mice had many more new cells than the wild-type ones at 24 h (Figure 4C), after 28 days, AD mice had fewer new cells, resulting in a much lower survival ratio (Figure 4D). However, treadmill exercise significantly increased new cell survival in both AD and WT mice (*p* < 0.01). Overall, APP and PS1 overexpression reduced the survival of new cells, and treadmill exercise notably reversed this influence.

#### 2.3.3. Treadmill Exercise Promoted Differentiation of Neurons and Suppressed Differentiation of Astrocytes

**Figure 5:**
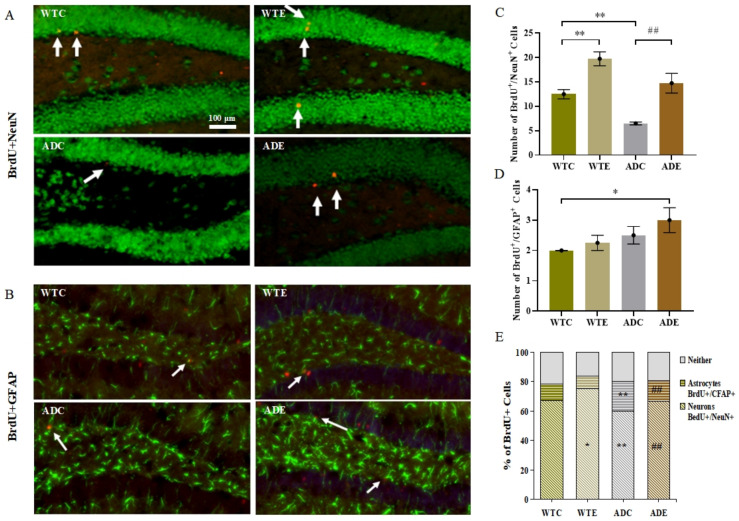
Effect of treadmill exercise on the differentiation of new cells in mice. (**A**) Fluorescence staining images of BrdU/NeuN-positive cells in 6-month-old mice. (**B**) Fluorescence staining images of BrdU/GFAP-positive cells in 6-month-old mice. (**C**) Number of BrdU/NeuN-positive cells in 6-month-old mice. (**D**) Number of BrdU/GFAP-positive cells in 6-month-old mice. (**E**) Differentiation rate of BrdU/NeuN- and BrdU/GFAP-positive cells. *n* = 4 for each group. * *p* < 0.05, ** *p* < 0.01 compared with WTC. ## *p* < 0.01 compared with ADC. The arrows point to the BrdU/NeuN co-localization-positive cell in (**A**) and BrdU/GFAP co-localization-positive cells in (**B**).

Four weeks after the last BrdU injection at week 8, we performed immunofluorescent double-labeling of BrdU/NeuN for neurons and BrdU/GFAP for astrocytes to evaluate the phenotypic differentiation of surviving cells (Figure 5A,B). Figure 5C shows the BrdU/NeuN-marked neurons count. Two-way ANOVA suggested that both genotype (F(1,12) = 16.98, *p* = 0.0014) and exercise (F(1,12) = 33.72, *p* < 0.0001), but not their interaction (F(1,12) = 0.1404, *p* = 0.7145), had significant main effects. The post-hoc analysis revealed that APP/PS1 transgenic mice had fewer BrdU/NeuN co-localization-positive cells than wild-type mice. Treadmill exercise significantly increased the number of neurons in both APP/PS1 transgenic and wild-type mice (*p* < 0.01). As shown in Figure 5D, astrocytes yielded different results. Only the genotype (F(1,12) = 5.0, *p* = 0.0451) had significant main effects. Exercise and APP overexpression both promoted astrocyte differentiation, albeit not significantly. Altogether, a high proportion of the total BrdU-positive cells were neurons, and the fraction of BrdU-positive cells not double-labeled was similar in the four mouse groups (Figure 5E). Thus, the ADC group had a significantly lower proportion of neurons than the WTC group (*p* < 0.01) but had notably more astrocytes (*p* < 0.01). Similarly, treadmill exercise significantly increased the fraction of neurons in the ADE group (*p* < 0.01) compared with the ADC group, and it prominently reduced the astrocytes to BrdU-positive cells ratio (*p* < 0.01). These results indicated that APP and PS1 overexpression also suppressed neuronal differentiation, and treadmill exercise markedly reversed this influence.

### 2.4. Effects of Treadmill Exercise on the APP Proteolytic Pathway

**Figure 6:**
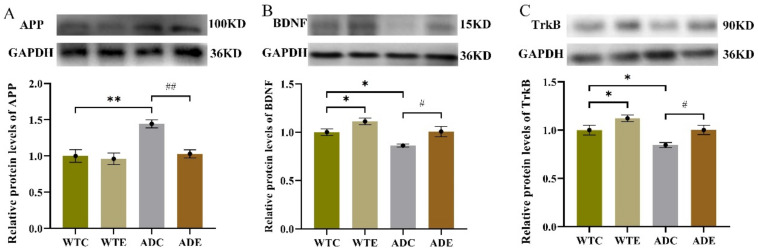
Effect of treadmill exercise on various protein levels in mice. (**A**) Relative APP protein levels in the hippocampus. (**B**) Relative BDNF protein levels in the hippocampus. (**C**) Relative TrkB protein levels in the hippocampus. *n* = 6 for each group. * *p* < 0.05, ** *p* < 0.01 compared with WTC. # *p* < 0.05, ## *p* < 0.01 compared with ADC.

**Figure 7:**
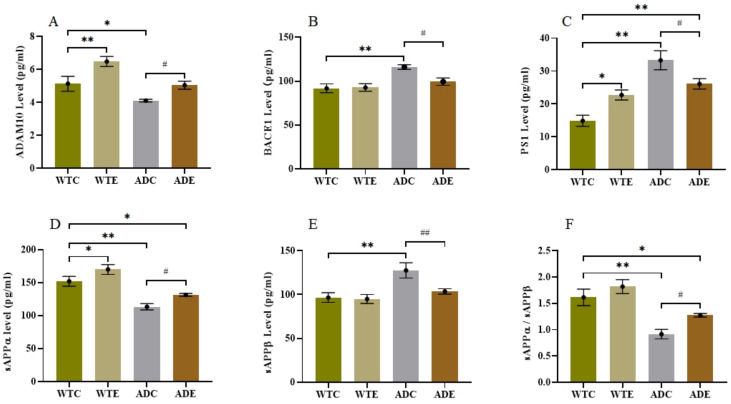
Effect of treadmill exercise on various secretase levels and APP metabolite production in mice. (**A**) ADAM10 levels in the hippocampus. (**B**) BACE1 levels in the hippocampus. (**C**) PS1 levels in the hippocampus. (**D**) sAPPα levels in the hippocampus. (**E**) sAPPβ levels in the hippocampus. (**F**) sAPPα/sAPPβ ratio. *n* = 6 for each group. * *p* < 0.05, ** *p* < 0.01 compared with WTC. # *p* < 0.05, ## *p* < 0.01 compared with ADC.

To investigate the influence of aerobic exercise on AHN in the APP/PS1 transgenic mice from a molecular perspective, we next quantified indicators related to APP metabolism. We determined the protein expression levels of APP, BDNF, and TrkB by Western blotting (Figure 6) and quantified ADAM10, BACE1, PS1, SAPPα, and SAPPβ in the hippocampus by ELISA (Figure 7).

As shown in Figure 6A, the post-hoc analysis revealed that the APP peptide level was remarkably higher in the ADC group than in the WTC group (*p* < 0.01), but it was significantly lower in the ADE group (after 3 months of treadmill exercise) than in the ADC group (*p* < 0.01). These were similar with Aβ peptide expression levels (Figure 2B) and displayed the pathological features of AD. The two-way ANOVA on the BDNF and TrkB protein levels in the hippocampus (Figure 6B,C) indicated that both genotype (*p* < 0.01) and exercise (*p* < 0.01), but not their interaction (*p* > 0.5), had significant main effects. The post-hoc analysis revealed that APP/PS1 transgenic mice had lower BDNF and TrkB protein levels than wild-type mice (*p* < 0.05). Treadmill exercise significantly increased the BDNF and TrkB protein levels in both APP/PS1 transgenic and wild-type mice (*p* < 0.05).

In the APP/PS1 mouse model, APP is a precursor protein of Aβ, and three key secretases, that is, ADAM10 (α-secretase), BACE1 (β-secretase), and PS1 (γ-secretase), are known to play an important role in its proteolytic process. As shown in Figure 7A, the WTC group had significantly less ADAM10 in the hippocampus than the WTE group (*p* < 0.01) and notably more than the ADC group (*p* < 0.05). Moreover, the ADE group had much higher ADAM10 levels than the ADC group (*p* < 0.05). As presented in Figure 7B,C, the BACE1 and PS1 levels were significantly higher in the ADC group than in the WTC group (*p* < 0.01) and remarkably lower in the ADE group than in the ADC group (*p* < 0.05). These results indicated that the amyloidogenic pathway was more active in the ADC group than in the ADE group, which was confirmed by the APP metabolite levels shown in Figure 7D–F. The ADC group had lower sAPPα and higher sAPPβ levels than the WTC group, resulting in a much lower sAPPα to sAPPβ ratio (*p* < 0.01). However, treadmill exercise improved the sAPPα/sAPPβ ratio significantly (*p* < 0.05) in AD mice, indicating that exercise could turn the amyloidogenic pathway into a non-amyloidogenic pathway.

## 3. Discussion

This study investigated the influence of 12-week treadmill exercise on AHN in APP/PS1 double transgenic mice and explored its APP proteolytic pathway-related molecular mechanism. We first demonstrated that AD mice had severe physiological and behavioral brain impairments and proved that exercise could reduce Aβ deposition and notably improve cognitive and learning abilities. Furthermore, the learning and memory deficits were found to be consistent with the sharp reduction in new neurons in the hippocampus of AD mice because new adult neurons contribute to hippocampus-dependent functions, such as memory and mood regulation [8]. Interestingly, besides increasing NSC proliferation, treadmill exercise promoted new cell survival and neuronal differentiation in 6-month-old AD mice. A further analysis of proteins and APP metabolism-related indicators revealed that an exercise-induced non-amyloidogenic APP pathway likely promoted AHN in mice.

Numerous reports have already confirmed that the hippocampus plays a critical role in learning and cognitive development in the adult brain [33], and AHN reduction or suppression leads to functional deficit [11]. Furthermore, increasing neurogenesis has increased the number of new neurons integrated into the neuronal network, partially improving information processing efficiency [8]. Herein, consistent with these conclusions, we showed that AD mice had fewer new neurons and performed poorly in the Morris water maze test, while mice that exercised had more neurons and performed significantly better (Figure 1, Figure 5C). Extensive evidence suggests that exercise promotes not only individual health but also brain functions related to cognition, reading, and attention [8,14,34]. Physical exercise prominently improved NPC proliferation in the DG [15] and enhanced spatial memory [35]. Furthermore, treadmill running above the lactate threshold could significantly promote hippocampal neurogenesis, and even treadmill exercise below the lactate threshold increased new neuron survival and maturation and improved spatial memory as well [16].

Consequently, intense physical exercise is beneficial for the proliferation, survival, differentiation, and maturation of cells in AHN, which our work further confirms (Figure 3, Figure 4 and Figure 5). Specifically, the increased proliferation observed in AD mice might be a stress reaction and an early pathology sign, while the exercise-induced increase in proliferation may be healthy cell growth. Relatively speaking, the decrease in proliferation and differentiation capacity in APP transgenic mice might attribute to that, in later-stage AD, NPC proliferative function is seriously impaired, and the hippocampal microenvironments are harsh. The next paragraph discusses the APP-related molecular mechanisms underlying exercise-induced AHN in AD mice.

NPCs derived from a non-neurogenic zone showed self-renewal and multi-potentiality when transplanted into a neurogenic niche in the brain and could differentiate in a region-specific context, which suggested that the microenvironment of the subgranular zone and subventricular zone plays a crucial role in NPC differentiation, survival, and differentiation by providing and modulating specific factors in the hippocampus [24,36]. As is well-known, the APP proteolytic pathway significantly influences the hippocampus microenvironment and the plasticity of adult brains [16]. Herein, AD mice had low BDNF and TrkB protein levels, but the mice who performed 12-week treadmill exercise had significantly higher levels of these proteins than the sedentary groups (Figure 6), which was consistent with other studies [37]. Moreover, BDNF can promote APP proteolysis in a non-amyloidogenic pathway by altering ADAM10 redistribution at the cell membrane [38].

Secretases are essential in determining the proteolytic pathway of APP. Under normal physiological conditions, the non-amyloidogenic pathway initially produces the α-C-terminal and sAPPα fragments in the cell membrane. Then, γ-secretase cleaves the α-C-terminal fragment and releases the P3 and AICD fragments, thereby preventing Aβ peptide formation and aggregation. Alternatively, under pathological conditions, β-secretase cleaves APP, generating the β-C-terminal fragment, which is then further cleaved by γ-secretase, releasing Aβ peptides and also the AICD fragment [39]. Thus, the amounts of various secretases can alter the APP proteolytic pathway. By quantifying three key secretases, we found that AD mice had low ADAM10 levels and high BACE1 and PS1 levels. Moreover, treadmill exercise significantly reversed this situation (Figure 7A–C). Consequently, the amyloidogenic pathway was predominant in AD mice, and treadmill exercise could promote APP cleavage through the non-amyloidogenic pathway. These results were consistent with the amounts of sAPPα and sAPPβ produced by APP metabolism (Figure 7D–F). Due to the levels of three key secretases, the APP cleaved more in amyloidogenic protein pathway than non-amyloidogenic pathway in AD mice, which led to generate much more sAPPβ and less sAPPα comparing to WT mice as shown in Figure 7D,E.

Furthermore, the non-amyloidogenic pathway induced by treadmill exercise helps to create a favorable hippocampal microenvironment with more neurogenesis, less toxic Aβ deposition, and neuroglia, which improves neuronal survival and differentiation, as shown in Figure 5 and Figure 6. Overall, we speculate that treadmill exercise first regulates neurotrophic factors and key secretases levels at early-stage AD, promoting APP cleavage by the non-amyloidogenic pathway. These effects enhance the hippocampal microenvironment, further improving new cell survival and neuronal differentiation, and finally alleviating the cognitive disorder. Understanding how the various neurotrophic factors and secretases might impair AHN at early-stage AD requires further exploration.

## 4. Materials and Methods

### 4.1. Animals and Experimental Design

We purchased 3-month-old male APP/PS1 ∆E9 mice on a C57BL/6J background and their wild-type C57BL/6J littermates from the Model Animal Research Center of Nanjing University (Nanjing, China). We then housed all the mice in ventilated cages and provided them with standard water and food ad libitum. We kept the dwelling environment at 22–24 °C and 50–60% humidity, under a 12 h/12 h light/dark cycle. After 2 days of adaptive feeding, we randomly assigned the mice to four groups, that is, the wild-type control group (WTC, *n* = 18), wild-type exercise group (WTE, *n* = 18), APP/PS1 control group (ADC, *n* = 18), and APP/PS1 exercise group (ADE, *n* = 18). We conducted the whole experiment in strict accordance with the relevant regulations required by the Experimental Animal Care and Use Committee at East China Normal University.

### 4.2. Treadmill Exercise Protocols

As we have previously described [31,40], the exercise program consisted of two stages. First, the ADE and WTE groups underwent adaptive training for 3 days. The exercise speed and time were 5 m/min for 10 min (day 1), 8 m/min for 20 min (day 2), and 12 m/min for 20 min (day 3). Second, the 3-month formal training period began after 2 days of rest, with an intensity of 45 min/day, 5 days/week. The daily training time was 18:00–20:00, and the detailed exercise scheme was as follows: the WTE and ADE groups obtained the same exercise protocols, they first exercised for 5 min at 5 m/min and 8 m/min, respectively; then, the speed increased to 12 m/min for 30 min, and it finally ended at 5 m/min for 5 min. Meanwhile, we placed the WTC and ADC group mice on a stationary treadmill.

### 4.3. 5-Bromo-2′-deoxyuridine (BrdU) Injections

We dissolved BrdU in 0.9 % normal saline at a 10 mg/mL concentration below 4 °C. A total of 12 mice would obtain BrdU injection. After 8 weeks of exercise, eight mice of each group received a daily BrdU injection (50 mg/kg) for 7 days. We processed the mice for BrdU immunostaining 24 h (four mice) and 28 days (four mice) after the last BrdU injection to assess NPC survival and differentiation rate. After the 12th exercise training week, four additional mice in each group received the same BrdU injection scheme and were processed 24 h after the last BrdU injection to assess NPC proliferation.

### 4.4. Morris Water Maze Test

The Morris water maze assay is a common way to evaluate spatial learning and memory function in transgenic mice [31,41]. After 12 weeks of exercise training, we selected six mice from each group for the Morris water maze test in order to assess their spatial learning and memory capacity. The Morris water maze consists of a pool (120 cm in diameter, 50 cm in height), a platform (8 cm in diameter), and a video capture system. The pool was divided into four equal quadrants and filled with water (containing milk) controlled at 24–26 °C. For the first 5 days, the platform was placed 1 cm underwater in the first quadrant of the pool. We then placed the mice into the water and recorded the time they spent escaping the water. We placed the mice who could not find the platform within 60 s on the platform for 10 s to promote learning. On the 6th day, we removed the platform and placed the mice into the water. We recorded the number of platform crossings and the total swimming distance within 60 s.

### 4.5. Tissue Preparation

We anesthetized the mice deeply by intraperitoneal injection of 2% pentobarbital sodium and perfused the 12 mice who had received BrdU with 0.1 M phosphate buffered saline (PBS, pH 7.4) transcardially, then followed by 4% paraformaldehyde (PFA) in PBS. After decapitating them, all brains were fixed overnight in 4% PFA, and dehydrated with 30% glucose for at least 24 h. Finally, the brains were frozen in embedding medium at −80 °C and cutted as cryosections (30-μm-thick) for staining experiments.

Twelve hours after the behavioral experiment, we decapitated the remaining six mice in each group and immediately removed the hippocampal tissues, which were frozen in liquid nitrogen and then stored at −80 °C for protein extraction for Western blotting and Enzyme-linked Immunosorbent Assay (ELISA). For frozen tissue, lysates were prepared utilizing a radioimmunoprecipitation assay buffer including 150 mM NaCl, 50 mM Tris (pH 7.4), 1% Triton X-100, 0.1% SDS, 2 mM EDTA and 1% sodium deoxycholate with protease-inhibitor cocktail tablet and phosstop phosphatase inhibitor cocktail tablet. We loaded hippocampal tissue (20 mg) in a homogenate tube with four to five magnetic beads and 1 mL radioimmunoprecipitation assay buffer, then mechanically homogenized them at 3.55 m/s for 30 s, and put on ice for 5 min. After repeating this process three times, the final homogenate was centrifuged at 4 °C for 10 min at 12,000 *g*. we collected the supernatant and applied the BCA method to determine the protein concentration.

### 4.6. Detection Methods

#### 4.6.1. Detection of Aβ by Thioflavin-S Staining

We detected Aβ using thioflavin-S staining as previously described procedures [32]. The cerebral cryosections were retrieved and washed by cold PBS (7.4) for three times, and then incubated with 0.3% thioflavin-S (T1892-25G, Sigma-Aldrich, Burlington, MA, USA) in the dark at ambient temperature for 8 min. After the slices were washed by cold PBS (PH 7.4) for three times, DAPI was utilized to stain nucleus. We used the Olympus VS120 microscope to capture the images of the brain slices.

#### 4.6.2. Detection of Apoptosis by TUNEL Assay

We let the prepared samples thaw for 20 min; then, we washed them with phosphate-buffered saline (PBS) twice. We added 50 μL of TUNEL mixture to the samples and then washed them again with PBS three times for 4′,6-diamidino-2-phenylindole (DAPI) dyeing. Finally, after rinsing the slices with PBS, we dried and sealed them with anti-fluorescence quenching sealing tablets.

#### 4.6.3. Immunofluorescence Staining

We used immunofluorescence staining to detect the cells marked by BrdU, BrdU/PSA-NCAM, BrdU/Neun, and BrdU/GFAP. We washed the prepared samples with TBS before and after acidifying them. We then incubated the slices with the primary antibody at 4 °C overnight and with the secondary antibody at 37 °C for 2 h. Next, we dyed the core with DAPI (1:3000) for 3 min. After washing and drying the slices, we sealed them with anti-fluorescence quenching sealing tablets.

In the above experiments, we used the following staining antibody dilution ratios: BrdU (Accurate Chemical, OBT0030, 1:100), PSA-NCAM (Sigma, MAB5324, 1:500), NeuN (Abcam, ab177487, 1:300), and GFAP (Abcam, ab10062, 1:1000), Dylight488 (Jackson Immuno Research, 711-485-152, 1:500), Dylight549 (Jackson Immuno Research, 711-505-152, 1:500).

#### 4.6.4. Western Blotting

We performed Western blotting as we have previously described [31,32]. We extracted the proteins from hippocampal tissues with a RIPA lysis buffer and determined the total protein concentration using a BCA protein assay kit (Sangon, Shanghai, China). Then we performed loading, electrophoresis, membranes transfer, closure, and incubation with the primary antibody overnight and the secondary antibody for 2 h. More specifically, for western blot analysis, protein samples (30 μg) were electrophoresed on 10% SDS-PAGE gels and transferred to a PVDF membrane in Tris-glycine buffer. We utilized the primary antibodies BDNF (Abcam, ab108319, 1:2000), TrkB (Cell Signaling, 4603, 1:1000), APP (Cell Signaling, 2452, 1:1000) in TBST overnight at 4 °C. GAPDH (Abcam, ab181602, 1:10,000) was used as the internal control. We incubated the blots with HRP-conjugated secondary antibodies and detected signals utilizing the enhanced chemiluminescence (ECL) WB detection reagents. The integrated density value (IDV) of the protein bands were quantified utilizing Alpha Ease FC software (GIS-2008; Tanon, Shanghai, China), and values were normalized with loading control.

#### 4.6.5. Enzyme-Linked Immunosorbent Assay

We performed the ELISA assay as we previously described [31,32]. We extracted the supernatant from the right hippocampal homogenate to quantify ADAM10 (ml241740, Mlbio, Shanghai, China), BACE1 (ml037908, Mlbio, Shanghai, China), PS1 (ml100603, Mlbio, Shanghai, China), SAPPα (ml127899, Mlbio, Shanghai, China), and SAPPβ (ml057880, Mlbio, Shanghai, China) following the instructions of the manufacturer of the ELISA kit. We then detected the absorbance at 450 nm using a spectrophotometer (TECAN Infinite M200, TECAN, Männedorf, Switzerland).

### 4.7. Graph Acquisition and Cell Counting

We captured all the data using an Olympus VS120 microscope. The indicators of the staining samples included Aβ plaque, TUNEL, BrdU, BrdU/PSA-NCAM, BrdU/NeuN, and BrdU/GFAP-positive cells. The detailed counting approach was as follows: we selected three brain slices with similar anatomical locations and counted all the cells in the left and right hippocampal regions. For each mouse, we averaged the counts from the three brain slices, and each group included at least four mice.

### 4.8. Statistical Analysis

We analyzed all the data using Graph Pad Prism 8.0 and expressed the results as the mean ± standard error of the mean. We compared the groups using two-way analysis of variance (ANOVA). We compared behavior performance in the Morris water maze test using repeated measures ANOVA. We performed post-hoc *t*-tests to further investigate statistically significant ANOVA results. We considered *p* < 0.05 as statistically significant.

## 5. Conclusions

In conclusion, 12 weeks of treadmill exercise promoted APP cleavage through a non-amyloidogenic pathway, improved the hippocampal microenvironment, and enhanced AHN in AD mice, thus improving their learning and memory capacity. These findings indicate that enhancing hippocampal neurogenesis by physical exercise may achieve hippocampal protection and become a therapy for the prevention and improvement of AD.

## Data Availability

Data that contained within the paper are available from the authors upon reasonable request.
